# Forkhead box transcription factor 1: role in the pathogenesis of diabetic cardiomyopathy

**DOI:** 10.1186/s12933-016-0361-1

**Published:** 2016-03-08

**Authors:** Vidya Kandula, Ramoji Kosuru, Haobo Li, Dan Yan, Qiqi Zhu, Qingquan Lian, Ren-shan Ge, Zhengyuan Xia, Michael G. Irwin

**Affiliations:** Department of Anesthesiology, The University of Hong Kong, Hong Kong, China; Department of Anesthesiology, The Second Affiliated Hospital and Yuying Children’s Hospital of Wenzhou Medical University, Wenzhou, Zhejiang China

**Keywords:** Diabetic cardiomyopathy, FOXO1, Metabolism, Oxidative stress, Inflammation, Apoptosis

## Abstract

Diabetic cardiomyopathy (DCM) is a disorder of the heart muscle in people with diabetes that can occur independent of hypertension or vascular disease. The underlying mechanism of DCM is incompletely understood. Some transcription factors have been suggested to regulate the gene program intricate in the pathogenesis of diabetes prompted cardiac injury. Forkhead box transcription factor 1 is a pleiotropic transcription factor that plays a pivotal role in a variety of physiological processes. Altered FOXO1 expression and function have been associated with cardiovascular diseases, and the important role of FOXO1 in DCM has begun to attract attention. In this review, we focus on the FOXO1 pathway and its role in various processes that have been related to DCM, such as metabolism, oxidative stress, endothelial dysfunction, inflammation and apoptosis.

## Background

Diabetic cardiomyopathy (DCM) is causative for 80 % of the fatality rate in the diabetic inhabitants [1, 2]. The molecular theory of DCM describes that, hyperglycaemia is the core pathogenic cause, which roots irregularities at the cardiac myocyte level, ultimately contributing to structural and functional anomalies [2]. This is also nurtured by the datum that patients with diabetes mellitus, independently of the viciousness of coronary artery disease, have amplified threat of heart failure in contrast with subjects deprived of diabetes mellitus [3, 4].

DCM linked pathologies have their ancestries in shifts of gene expression. Although various origins of cardiac injury have been identified, the vital molecular contraptions of their pathogenesis have not been anticipated. The molecular operations within the cardiac cell are organised by transcription factors such as forkhead box (other) transcriptional factor (FOXO). In heart diseases such as atherosclerosis [5], diabetic cardiomyopathy [6, 7], there is an augmented FOXO activity. In this paper we will focus on the possible role of FOXO regulation in DCM. This entity embraces the direct outcome of diabetes mellitus, as one of the utmost rampant diseases, on the myocardium. FOXO regulation may deed in DCM via numerous pathways, by governing different set of genes being involved in allied processes such as oxidative stress, metabolism, inflammation, endothelial dysfunction and apoptosis.

## FOXO’s

The “forkhead” name was conferred on account of the two spiked-head structures observed in the embryos of the *Drosophila Melanogaster forkhead mutant* and its part was connected to the development of gut of the Drosophila fetus [8]. Forkhead proteins are identified as novel class of transcription factors in the late 20th century [9]. FOXO is one amongst 19 families of FOX superfamily and incorporates FOXO1, 3, 4, and 6 [10, 11]. FOXO in humans is similar to dFOXO in *Drosophila Melanogaster*, and abnormal dauer formation-16 in *Caenorhabditis elegans* [10].

FOXO’s consists of highly conserved forkhead/winged helix DNA-binding domain, which encompasses the most common 110 amino acids of FOXO family and embodies 3a, 3b, and 2 winged helices, facilitating its DNA binding [12, 13]. FOXO1 and FOXO3 are expressed globally, and FOXO1 isoform is abundantly located in hepatic, fatty tissue and pancreatic β cells [14, 15]. FOXO4 is mainly located in muscle, renal, and colorectal tissue while FOXO6 is predominantly located in the liver and cerebrum [16]. Post-translational modifications (PTM) such as phosphorylation, acetylation, ubiquitination, arginine methylation, and O-glycosylation [13, 17] are known to determine the FOXO1 nuclear transit and transcriptional activity [18]. These modifications can either enhance or reduce the FOXO1 transcriptional activity as determined by the upstream target and/or the sites concerned [17]. AKT phosphorylates FOXO1, facilitating its nuclear transit, which consecutively decreases the transcriptional function of FOXO1 [19–21]. However, several other kinases like mitogen-activated protein kinases (also known as JNKs), cyclin-dependent kinase 2 and nuclear factor κB (NFκB) kinase are also involved in FOXO1 phosphorylation [22–24]. The nuclear compartmentalization and transcriptional function of FOXO1 can also be modified by other PTM like acetylation, ubiquitina-tion, glycosylation and methylation [25–29].

Over the recent decade, numerous studies have uncovered the essential functions of FOXOs in managing diverse range of cellular processes. FOXO1 is the key member among the ‘O’ subfamily, in controlling equilibrium of cardiac cells [18, 30]. Global loss of FOXO1 is fatal as it initiates embryonic cell death because of inadequate vascular growth [31]. From the embryo to adulthood, FOXO factors play an important role in maintaining cardiac homeostasis [32]. Furthermore, FOXO1 is concerned in controlling cellular responses like oxidative stress response, cell multiplication, immune homeostasis, cell death, and metabolism in diverse kinds of tissues [33].

## FOXO regulated genes

In relation to the heart, FOXO controls the expression of a variety of target genes that are involved in cellular metabolism, oxidative stress, apoptosis and cell cycle differentiation (Table 1). Interestingly, FOXO factors have been shown to be regulated by numerous stress stimuli, including DNA damage, cytokines, nutrient and oxygen deprivation [24, 34–39]. In addition, stimulation of FOXO factors by 5′ adenosine monophosphate-activated protein kinase stimulates the preferential expression of a gene expression program that heightens cellular stress resistance [37, 38]. In spite of the fact that the regulation of FOXO components is majorly controlled by posttranslational changes, a series of latest studies have emphasized how FOXO factors additionally coordinate extracellular stimuli through substitute mechanisms. For instance, the growth regulatory cytokine such as transforming growth factor β triggers the expression of genes involved in cell cycle inhibition like p15 and p21 through complex formation between FOXO, Smad, and C/EBPb transcription factors at particular promoters [34–36, 40, 41]. These most recent studies highlight the complex regulation of the FOXO transcription factors, by an extensive variety of different stimuli, including cytokines, glucose availability, DNA damage and oxygen deprivation, that may aid to refine FOXO function in distinctive cell kinds under diverse environmental settings.

**Table 1:** Potential main stimuli and FOXO1 related genes involved in DCM

FOXO1 stimuli	FOXO1 targeted genes	Function	Reference
Insulin	↓Phosphoenolpyruvate carboxykinase (PEPCK) ↑Glucose-6-phosphatase (G6Pase) ↓Apolipoprotein C-III (apoC-III)	↓Gluconeogensis Promotes blood glucose homeostasis by ↑glucose production ↓Triglyceride levels	[42–44]
The Peroxisome Proliferator Activated Receptor (PPAR) γ Coactivator 1-α (PGC-1α)	↑Pyruvate dehydrogenase kinase 4 (PDK4)	↑Gluconeogenesis	[45]
Fructose Fibrates	↑apoC-III ↓apoC-III	↑Triglyceride levels ↓Triglyceride levels	[46]
High glucose	↑Thioredoxin interacting protein (Txnip) ↑Inducible nitric oxide synthase (iNOS)/Nitric oxide (NO) ↓Kruppel-like factor 2 (KLF2) ↑B cell leukemia/lymphoma 2-associated death promoter (BAD)	↑Endothelial oxidative stress ↑Atherosclerosis ↑Endothelial dysfunction (impairment of endothelium-dependent vasodilatation) ↑Cell death	[5, 47–49]
Vascular endothelial growth factor (VEGF)	↑Manganese superoxide dismutase (MnSOD), ↑Bone morphogenic protein 2 (BMP2), ↑Vascular cell adhesion molecule (VCAM-1), ↑Matrix metalloproteinase-10 (MMP10)	↑Endothelial cell survival and proliferation	[50]
Reactive oxygen species (ROS)	↑iNOS/NO	↓Endothelial function	[5]
Pressure overload, oxidative stress, sirtuin1 (SIRT1)	↑Catalase	↓Oxidative stress (Impaired ROS homeostasis)	[51]
Hydrogen peroxide (H_2_O_2_)	↑Bim (Proapoptotic factor)	↑Oxidative stress in endothelia cells	[52]
Tumor necrosis factor- α (TNF-α)	↑CCAAT/enhancer binding protein (C/EBPβ)	↑Production of proinflammatory cytokines like monocyte chemoattractant protein (MCP)-1, interleukin(IL)-6	[53]
Insulin-like growth factor-1, angiotensin-II	↑Modulatory calcineurin interacting protein exon 4 isoform (MCIP1.4)	↑Cardiac hypertrophy	[54]
Poly(ADP-ribose)polymerase-1 (PARP-1)	↓Cell cycle inhibitor p27(kip1)	↑Cell proliferation	[55]
Endothelin-1 (ET-1)	↓BAD	↓Cell apoptosis	[49]
Resveratrol	↑Rab7	↑Myocardial autophagic flux	[56]

Different stimuli can activate or suppress FOXO1 signalling and several targeted genes may be controlled by FOXO1 in the diabetic conditions

## The role of FOXO1 in the heart

Among the FOXO subfamily, FOXO1, FOXO3, FOXO4 are expressed in the heart [57] and localizes in nucleus where they get associated with coactivators and modulate multiple signal transduction pathways [34, 58–60]. The impacts of FOXO family on heart function and cardiac remodelling have been reviewed [61, 62]. Within the setting of cardiac function, the FOXO proteins are supposed to be participated in oxidative stress [63], regulation of metabolism [58], cell cycle [64], and cell death [65]. Fetal development of heart obliges cell development and division, and FOXO1 is essential for embryonic vascular and cardiac development. For instance, FOXO1 gene knocked out embryos die at E10.5–E11 day and demonstrated curbed vascular and cardiac growth [31, 66]. On the contrary, mice overexpressing a myocardial specific FOXO1 also die by E10.5 because of anomalous expression of cyclin-dependent kinase inhibitors p21 (cip1) and p27 (kip1), resulting in diminished proliferation of cardiomyocytes, decreased cardiac size and myocardium thickness, and ensuing cardiac catastrophe [67]. Myocardial SIRT1 overexpression and precludes aging of heart via FOXO1 mediated induction of catalase expression [51].

## FOXO1 and cardiovascular diseases

Dysregulated activity of FOXO1 has been implicated in the pathophysiology of DCM [6, 7, 68], ischemic heart disease [69] and cardiac hypertrophy [54]. In general, FOXO1 has been found to play a protective role in ischemic heart diseases. For instance, Benzhi Cai et al. [70], reported that deletion of FOXO1 in heart caused an increment in myocardial Na+ load by augmenting NaV1.5, a principle α subunit of the cardiac sodium ion channel and Na+ channel subunit β3 mRNA and resulted in shortening of QRS complex significantly, proposing that FOXO1 is facilitating the modulation of sodium channel in ischemic cardiomyopathy. The protective role of FOXO1 is further confirmed by Sengupta et al. who observed that in mice with cardiac-specific deficiency of both FOXO1 and FOXO3 the hearts demonstrated lowered systole, enlarged scar development and amplified apoptosis relative to control mice, when subjected to myocardial infarction through surgical ligation of coronary artery [71]. Moderate increment of FOXO1 expression in heart diminished while its cardiac overexpression in diabetes [72] or deficiency aggravated myocardial ischemia reperfusion injury [71]. The role of FOXO1 in mediating diabetic heart susceptibility to ischemia–reperfusion injury has recently been reviewed by our group [72]. Besides, in light of an ischemia–reperfusion convention, the FOXO1 and FOXO3 dual knockout mice displayed diminished expression of catalase and Manganese superoxide dismutase (MnSOD). Furthermore, both FOXO1 and FOXO3 transcription factors prevent cardiac hypertrophy by stimulating the expression of atrogin-I (an E3 ubiquitin ligase) that facilitates the inhibition of calcineurin/nuclear factor of activated T cells [54]. In addition, ubiquitinization of FOXO1 by atrogin-I promote its nuclear retention and enhancement of its transcriptional activity, and facilitate to oppose the Akt-dependent physiological hypertrophy [54, 73]. In contrast to the protective role, Yajuan Qi et al. [6] recently observed that FOXO1 plays a prominent role in the development of DCM. FOXO1 upregulation in insulin resistance state may lead to impairment of cardiac contractility by increasing β-myosin heavy chain gene expression in cardiac cells [6].

## FOXO1 and DCM

**Fig. 1:**
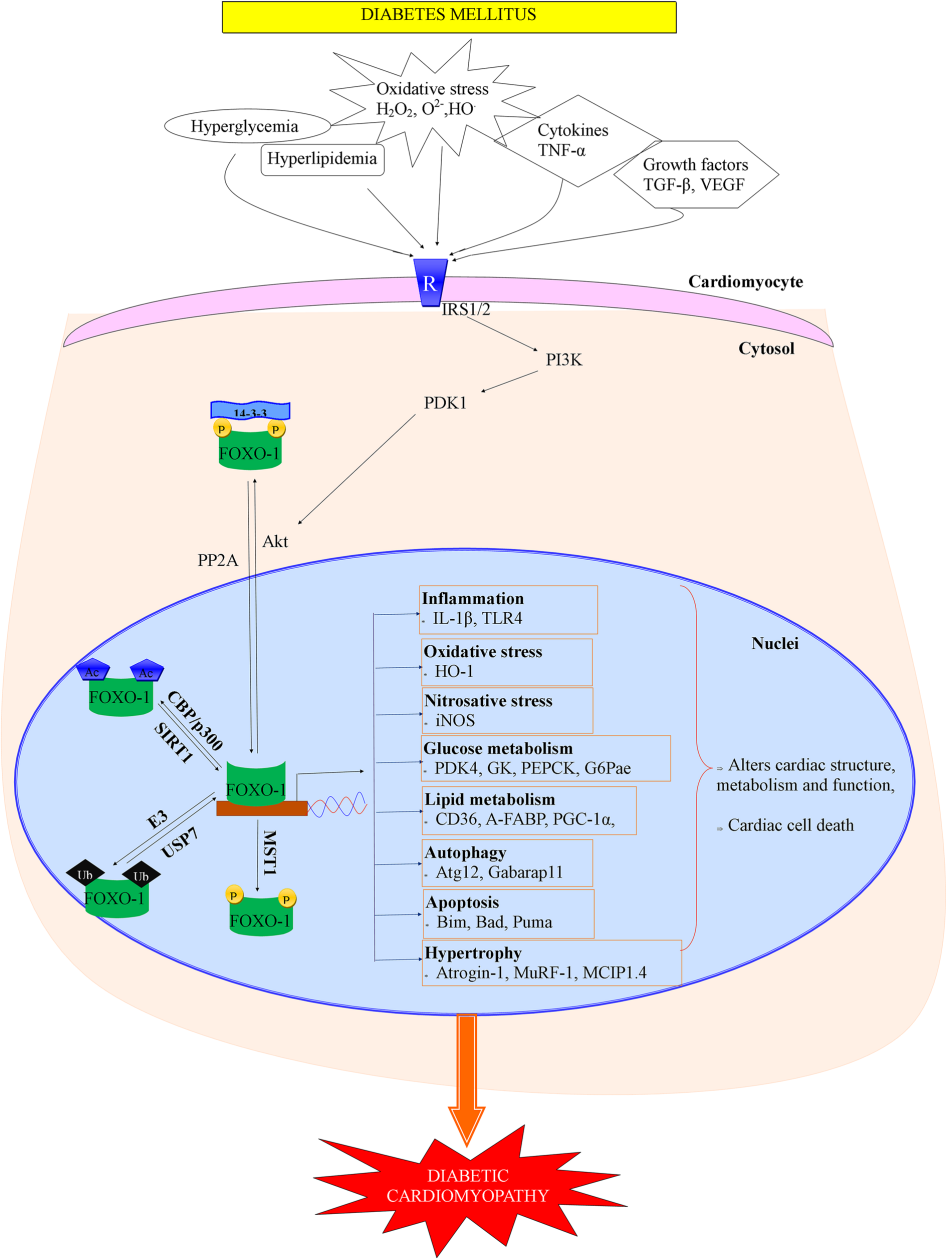
Regulation and function of FOXO-1 in the development of DCM. In diabetes mellitus, various stimuli like excess glucose, excess lipids, oxygen free radicals, cytokines and other growth factors triggers several mechanisms that promote posttranslational modifications like phosphorylation, acetylation, deacetylation which may regulate the FOXO-1 activity and function. Akt promotes the phosphorylation and translocation of FOXO1 to cytosol and facilitates its binding with 14-3-3 protein which directs it for degradation whereas protein phosphatase 2A (PP2A) causes dephosphorylation and translocates FOXO1 to nucleus from cytosol. E3 ubiquitin ligase facilitates ubiquination of FOXO1, while ubiquitin specific protease (USP7) inverted the process. Further, phosphorylation by Mst1 stimulates FOXO1 transcriptional activity. In addition, cAMP response element binding protein (CBP) and p300 histone acetyltransferase acetylates FOXO1, and silent information regulator 1 (SIRT1) deacetylates it. Activated FOXO-1 binds to the FOXO-binding site and triggers several genes involved in inflammation, oxidative stress, nitrosative stress, glucose and lipid metabolism, hypertrophy, autophagy and apoptosis that finally leads to alteration of cardiac structure, metabolism, function and cardiac cell death. *P* phoshorylation; *Ub* ubiquitination; *Ac* acetylation

Diabetic patients are more prone to the risk of cardiomyopathy, and heart failure is a foremost cause of death in diabetes populations [74–76]. This could not be explicated by considering several other diabetes related risk factors such as dyslipidemia, obesity, infarction, endothelial dysfunction. Thus, diabetes mellitus independent of coronary vascular disease and hypertension can modify the structures as well as functions of the myocardium, a state acknowledged as DCM [77]. In this process, the metabolic derangements in glucose and lipids trigger rigorous cardiac changes that progresses to dysfunction of ventricular diastole and systole [78]. Furthermore, lipid overload in the myocardium leads to contractile dysfunction in animal models and humans by upregulating gene expressions of peroxisome proliferator activated receptor (PPAR)α, myosin heavy chain (MHC)-β, and tumor necrosis factor (TNF)-α [79]. In addition, deficiency of muscle ring-finger protein (MuRF)2, an ubiquitin ligase resulted in the development of diabetic cardiomyopathy by enhancing cardiac PPARα and PPARγ1 gene expression [80]. However, cellular mechanisms associated with DCM, including FOXO1 signalling, are not yet completely understood [77]. Understanding for the pathogenesis of DCM is stemmed mainly from in vivo animal models [81, 82]. Recent in vivo and in vitro studies indicate that enhanced cardiac FOXO1 activation has been illustrated in diabetic mice [7]. The responses in the heart such as metabolic adaptation, oxidative stress, endothelial dysfunction, inflammation, and apoptosis in which FOXO1 could participate that may lead to DCM are illustrated below (Fig. 1).

### FOXO1 and DCM-associated metabolism

Disturbances in myocardial glucose and lipid metabolism are initial events that lead to cardiac dysfunction in diabetic condition. FOXO1 is involved in various pathways related to cellular energy metabolism. During insulin resistance, pyruvate dehydrogenase kinase 4 (PDK4) is known to inhibit glucose oxidation by blocking pyruvate to enter into mitochondrial oxidation through phosphorylating the E1 moiety of pyruvate dehydrogenase complex [83]. Enhanced expression of FOXO1 downstream target gene PDK4 gene is also observed in high fat and obese animal models of insulin resistance, which adversely regulate insulin actions [84, 85]. FOXO1 can modulate glucose metabolism in adult cardiomyocytes in insulin resistance and diabetic conditions by inhibiting glucose oxidation preceded by PDK4 activation, subsequently altering the substrate preference for fatty acid and lactate [33, 83]. In addition, FOXO1 upholds gluconeogenesis in liver by enhancing G6Pase and PEPCK mRNA [86, 87]. Besides, FOXO1 depletion abolished the high-fat diet induced aberration of glycolytic genes (declined hexokinase 1 and glucose transporter 4) and lipid oxidation gene expression patterns (diminished PGC-1α and amplified PDK4). These findings collectively suggest that FOXO1 is a key factor responsible for anomaly of glucose and lipid metabolic pathways in insulin resistance.

Surprisingly, Battiprolu and his colleagues noticed morphological and functional myocardial modifications similar to DCM in two dissimilar animal models of type 2 diabetes, db/db mice lacking leptin receptor and high-fat diet obese mice, as an outcome of FOXO boosted pathological alterations. Moreover, mice with cardiac specific FOXO1 knock out were resistant to high-fat diet provoked myocardial dysfunction and hypertrophy [7]. Recent evidences suggest that FOXO1 plays a key role in regulating cardiac aberrant metabolisms (i.e.; glucose and fatty acid metabolism) leading to DCM [7, 83]. These studies concludes that FOXO1 stimulation promote cardiac remodelling by altering cardiac metabolism whereas FOXO1 deletion resists cardiac remodelling. Thus, FOXO1 was distinguished as a central player in the cardiac metabolic abnormalities in DCM.

Another cardinal feature of DCM is lipotoxicity resulting in part from excessive lipid accumulation [79]. Puthanveetil et al. suggested that FOXO–iNOS–cluster domain 36 transporter (CD36) axis was involved in lipid accumulation in cardiomyocytes under lipid excess conditions. Oversupply of lipids augments the nuclear localization of FOXO1 accompanied by augmented CD36 translocation to the membrane without affecting its mRNA or total protein content, ensued by amplified lipid oxidation and triglyceride accrual [30]. Further, FOXO1 nuclear compartmentalization and enhancement of its transcriptional activity was observed especially during diabetes and obesity [88, 89]. Enhanced fatty acid flux into the cardiac cell can boost its oxidation with subsequent production of reactive oxygen/nitrogen species [90], which can subsequently induce hypertrophy and cardiac failure [91]. Additionally, FOXO1 has indispensable role in positive regulation of adipocyte fatty acid binding protein (FABP4) gene transcription, thereby controlling uptake and accumulation of lipids in macrophages, and promoting atherosclerosis [92]. Thus, FOXO1 over-activation plays a prominent role in myocardial lipid accumulation as a result of augmented lipid uptake over deployment.

Latest studies have disclosed the FOXO1 ability to relate insulin pathway to numerous kinds of metabolic stress in the heart. FOXO1 indirectly controls the insulin sensitivity via negatively modulating the insulin sensing genes. Insulin sensitivity is restored upon FOXO1 restricted deletion in genetic mouse model of insulin resistance, and this conditional deletion further reduced the expression of genes related to gluconeogenesis (e.g., G6pase and PEPCK1) in the liver and enhanced insulin sensitizing genes expression (e.g., Leptin gene, PPAR*γ*, and solute carrier family two (facilitated glucose transporter), member four) in adipocytes, thereby salvaged the diabetic phenotype [93]. Battiprolu et al. further demonstrated that FOXO mediated feedback control of insulin signalling played an essential role in DCM through inactivation of insulin receptor substrate 1 (IRS-1) [7]. In a preceding study, Niet et al. reported that FOXO stimulation promoted insulin resistance and impairment of glucose metabolism in primary cardiomyocytes through modulating Akt phosphorylation [94]. These observations of the FOXO-regulated vicious cycle of insulin resistance offer novel perceptions in the understanding of the pathogenesis of DCM and other diabetic complications.

The major role of FOXO factors in myocardial metabolic stress adaptation may be correlated through their regulation of autophagy [95, 96], a characteristic of numerous versatile responses of the cardiomyocyte, comprising the reactions to starvation, ischemia–reperfusion, and pressure overload [97, 98]. FOXO1 deacetylation by sirt1 (a class III histone deacetylase) seems to be an essential module of the autophagic reaction to nutrient deficit, and possibly different types of metabolic stress in cardiomyocytes [99]. In the heart, FOXO transcription factor can promote autophagy by stimulating autophagic genes such as Atg12 and Gabarapl1 under stress circumstances [100].

### FOXO1 and DCM-linked oxidative stress

Hyperglycemia and the subsequent formation of advanced glycation end-products are major culprits for the generation of ROS within the cardiac tissue in diabetes [101]. In addition, excess mitochondrial oxidation resulting primarily from lipid degradation is another cause for oxidative stress in cardiomyocytes [102]. ROS like hydroxyl, superoxide anion and H2O2 are extremely irritable and can trigger destruction to lipids, proteins and DNA [103]. As oxidative stress plays a significant role in the development of DCM [104], antioxidant therapy can be beneficial for the amelioration of DCM. Recently, rutin, a flavonoid antioxidant was shown to attenuate myocardial ventricular dysfunction and cardiac remodeling in diabetic condition [105]. Hyperglycemia provoked disruption of cellular protective antioxidant mechanisms has been associated in the progression of cardiovascular ailments. Xiaonan Li et al. reported that FOXO1 takes part in the mediation of high glucose induced elevation of oxidative stress that prompted the dysregulation of thioredoxin (Trx) antioxidant system [47]. Hyperglycemia promotes coupling of FOXO1 to the Txnip promoter that is facilitated by p38 MAPK pathway [47]. FOXO1 seems to stimulate cell demise, particularly in tissues that are influenced by diabetes associated complications where oxidative stress is beyond normal limits [106]. However, FOXO1 has an imperative role in cellular protection against oxidative stress by inducing enzymes such as MnSOD and catalase that catalyse ROS [63, 107]. FOXO1 can safeguard pancreatic* β*-cells against oxidative stress [108].

### FOXO1 and DCM-related endothelial dysfunction

Endothelial dysfunction is the harbinger of atherosclerosis that drives the detrimental consequence of diabetes on the heart. Endothelial dysfunction has been revealed in both type 1 and type 2 diabetic patients [109, 110] as well as in several animal models of diabetes [111]. The plausible mechanisms by which FOXO1 causes some features of endothelial dysfunction include the controlling of the expression of both isoforms of nitric oxide synthase enzyme i.e. inducible and endothelial isoforms. It is apparent that impaired endothelial nitric oxide synthase (eNOS) activity leads to endothelial dysfunction [112]. FOXO1 repress transcription of eNOS in endothelial cells [113]. In consistent with this, a recent study by Lee et al. showed that malfunction of FOXO1/KLF2/eNOS signalling promotes diabetic endothelial dysfunction [48]. Besides eNOS regulation, gain of function of FOXO1 in vascular endothelial cells further increased iNOS mRNA with resultant endothelial dysfunction [5]. This is in consistent with the finding that FOXO1 deletion in streptozotocin induced diabetic mice attenuated lipid peroxides and aortic iNOS activation in vascular endothelial cells.

Hyperglycemia-induced oxidative stress promotes post translational modifications of FOXO1 and its nuclear translocation, accounting for its enhanced transcriptional activity, thereby its participation in the progression of atherosclerosis in diabetic patients [5, 49]. Li et al., employed FOXO1 KR/KR knock-in mice to mimic the effect of oxidative stress- (or hyperglycemia-) induced FOXO1 deacetylation on atherosclerosis and demonstrated that FOXO1 gain of function in vascular endothelial cells trump its beneficial effects to lower triglycerides and low density lipoprotein cholesterol levels in its counterpart WTD-fed Ldlr-/- mice, suggesting that FOXO1 is involved in primary atherogenic abnormality occurred in the vascular endothelium [114]. Furthermore, unbridled activity of FOXO1, as a result of hyperglycemia induced O-glycosylation, accounted for its disproportionate regulation of apoptotic and proapoptotic factors (decreased Bcl-2 and increased caspase-3 and BAD), thereby contributing to endothelial cell death in human aorta endothelial cells [49]. In addition to this, any interruption in the endothelin (ET)1-Akt-FOXO1 feedback loop may be a contributing element for ET-1 deregulation and endothelial dysfunction in inadequately managed diabetes mellitus [49].

### FOXO1 and DCM-induced inflammation

Inflammation has deemed as a prime causative factor in diabetes and linked with the occurrence of heart failure in DCM. It has been reported that diverse stimuli like high glucose, TNF-α and lipopolysaccharides regulate the expression of proinflammatory cytokines via FOXO1 [106]. In insulin resistant obese mice model, macrophages have amplified FOXO1 stimulation with concomitant elevation of IL-1*β* production. Furthermore, FOXO1 accomplishes IL-1*β* expression by combining directly with the IL-1*β*promoter [115]. On the other hand, inflammatory cytokines stimulate FOXO1 and may be indulged in positive feedback loop. This was supported by the findings of Behl et al. who observed that augmented TNF-α in diabetes stimulated FOXO1 and this in turn further stimulated the expression of TNF-α levels in microvascular endothelial cells [88]. Elevated glucose levels in diabetes also activate toll-like receptor (TLR) pathway, which causes long-lasting inflammation and tissue injury. A recent study disclosed that FOXO1 supports inflammation during diabetes by increasing the expression of TLR4, recommending that FOXO1 may function as an essential regulator of inflammatory reactions during obesity and diabetes mellitus [116]. Since insulin is involved in FOXO1 repression, FOXO1 is stimulated in insulin resistance condition where diminution of insulin signalling pathway prevails, leading to accelerated inflammatory response. Furthermore, cell fate i.e. whether a cell experiences survival or apoptosis is governed by the relative balance between NF-κB and FOXO1, under inflammatory settings where both factors are triggered [117, 118]. This emphasizes the role of FOXO1 transcription factor as a central mediator of inflammation in the perspective of insulin resistance and obesity.

### FOXO1 and DCM-involved apoptosis

Cellular damage that progresses to apoptosis can be considered as an important contributing factor to pathology in maladies like diabetes and cardiovascular injury [119]. FOXO1 is the significant contributor in regulating cell death [33]. FOXO1 emerges as a potential regulator of different kinds of cell death during insulin resistance and diabetes. In diabetes, both intrinsic (mitochondrial cytochrome c mediated) and extrinsic (death receptors like Fas or TNF α mediated) apoptosis are reported to be augmented and FOXO1 is suggested to increase the expression of caspases and cell death receptors [120, 121]. Moreover, FOXO1 signalling can increase the proapoptotic gene expression like Bim and Puma [122, 123] and BAD [30]. Puthanveetil et al. suggested that FOXO1 regulates BAD, a pro-apoptotic factor through PP2A induction in in vivo models of diabetes and insulin resistance [30]. However, FOXO1 overexpression in cardiomyocytes suppresses the PP2A/B activity [94]. The discrepancy between the in vivo and in vitro models in relation to the effect of FOXO1 on PP2A could be the presence of excess lipids brought in by the FOXO1–CD36 pathway observed with diabetes, which turns on PP2A, thereby activating BAD stimulated apoptotic process.

### FOXO1 and DCM-associated mitochondrial dysfunction and calcium handling

Mitochondrial malfunction associated with insulin resistance is a prime contributing factor for DCM. FOXO1 is involved in the integration of mitochondrial function with insulin signalling. Elevated FOXO1 levels in insulin resistant states disrupt mitochondrial electron transport chain, thereby promoting impaired oxidative respiration [124]. In addition, FOXO1 is involved in the regulation of the mitochondrial biogenesis by affecting the expression of genes regulating mitochondrial fission and fusion through SIRT1/PGC1α pathway [124].

Stringent regulation of intracellular calcium homeostasis is essential for normal maintenance of cardiac function and growth [125]. Numerous challenges like accumulation of long-chain acetylcarnitines and oxidative stress can lead to impairment of calcium homeostasis in DCM [126]. DCM has been associated with altered function of sarco-endoplasmic reticulum Ca^2+^-ATPase, and Na+/Ca^2+^ exchanger (NCX) [127–129]. Altered calcium handling contributes to endoplasmic reticulum stress [130] and is the underlying cause for FOXO1 aggravation of diabetic cardiomyocyte cell death in response to ischemic insult [131]. In addition, enhanced levels of interleukin (IL)-1β contributed to endoplasmic reticulum stress induced DCM via IL-1 receptor-associated kinase-2/C/EBP homologous protein pathway [132]. It has been identified that calcium calmodulin-dependent kinase II has profound effect on FOXO1 nuclear retention, which may lead to excessive glucose production in the liver, in the context of obesity [133]. Surprisingly, in failing cardiomyocytes, enhanced cytoplasmic calcium levels helps facilitate calcium/calmodulin-dependent protein kinase dependent stimulation of Akt which leads to down regulation of microRNA-1 and NCX-1 expression by inhibiting FOXO3A activity [134]. Altogether, these studies implicate the role of FOXO1 in DCM associated mitochondrial dysfunction and calcium handling.

## Conclusions

In summary, FOXO1 regulation may contribute to the detrimental outcomes of the cardiac cells in diabetes, accelerating the development of DCM, one of the predominant cardiac difficulties in diabetic patients. Metabolic alterations, oxidative stress, endothelial dysfunction, inflammation and apoptosis have been shown to be implicated in the development and progression of DCM, and also in the desirable processes for the regulation of FOXO1 gene. Dysregulated FOXO1 expression and activity appear to promote endothelial dysfunction, myocardial oxidative stress, cardiomyocyte cell death and inflammation observed in DCM. Thus, FOXO1 or, favourably, any of its distinctive pathways may be of extreme concern for pharmaceutical target. However, mechanisms controlling the activity and expression of FOXO1 isoform in DCM are not well appreciated. There are so many unanswered questions relating to the FOXO1 activity in DCM. Some of them like, what could be the mechanistic link whereby FOXO1 activation contributes to the increased vulnerability of diabetic heart to ischemic insults? What could be the cause for persistent activation of FOXO1 in cardiac tissue in the settings of insulin resistance, lipid overload, elevated inflammatory cytokines and hyperglycemia? Whether FOXO1 plays similar roles like insulin resistance, in non-obese type 1 diabetic patients given that insulin deficiency is another characteristic feature of diabetes? Could FOXO1 gene polymorphism be responsible for individual susceptibility of DCM? What could be the direct metabolic consequences of FOXO1 activation? What is the functional role of FOXO1 in nonmyocytes of the heart? Thus, further research is necessary to unveil the precise mechanism of FOXO1 in the development and progression of DCM.

## Abbreviations

DCM: diabetic cardiomyopathy
FOXO: forkhead box (other) transcriptional factor
PTM: posttranslational modifications
NFκB: nuclear factor κB
PEPCK: phosphoenolpyruvate carboxykinase
G6Pase: glucose-6-phosphatase
apoC-III: apolipoprotein C-III
PPAR: peroxisome proliferator activated receptor
PGC-1α: peroxisome proliferator activated receptor γ coactivator 1-α
PDK4: pyruvate dehydrogenase kinase 4
Txnip: thioredoxin interacting protein
iNOS: inducible nitric oxide synthase
NO: nitric oxide
KLF2: kruppel-like factor 2
BAD: B cell leukemia/lymphoma 2-associated death promoter
MnSOD: manganese superoxide dismutase
BMP2: bone morphogenic protein 2
VCAM-1: vascular cell adhesion molecule
MMP10: matrix metalloproteinase-10
VEGF: vascular endothelial growth factor
ROS: reactive oxygen species
SIRT1: sirtuin1
H2O2: hydrogen peroxide
TNF-α: tumor necrosis factor- α
C/EBPβ: CCAAT/enhancer binding protein
MCIP1.4: modulatory calcineurin interacting protein exon 4 isoform
PARP-1: poly(ADP-Ribose)polymerase-1
ET-1: endothelin-1
CD36: cluster domain 36 transproter
A-FABP/FABP4: adipocyte fatty acid binding protein
IRS-1/2: insulin receptor substrate 1/2
Trx: thioredoxin
HO-1: heme oxygenase-1
eNOS: endothelium nitric oxide synthase
IL: interlukin
TLR: toll-like receptor
PP2A: protein phosphatase 2A
PDK1: 3-phosphoinositide dependent protein kinase-1
PI3K: phosphatidylinositol-3-kinase
TGFβ: transforming growth factor beta
MST1: mammalian homolog of Ser/Thr kinase 1
USP7: ubiquitin specific protease 7
CBP: cAMP response element binding protein
PUMA: p53 upregulated modulator of apoptosis
GK: glucokinase
MuRF: muscle ring-finger protein
ET-1: endothelin-1
MHC: myosin heavy chain
